# The effect of COVID-19 on medical student clinical skill practice and self-perceived proficiency

**DOI:** 10.12688/mep.19478.1

**Published:** 2023-02-08

**Authors:** Leanne Hall, Sophie Binks, Clare Heal

**Affiliations:** 1 College of Medicine and Dentistry, James Cook University, Mackay, Queensland, 4740, Australia

**Keywords:** COVID-19, medical education, clinical skills, proficiency

## Abstract

**Background: **The coronavirus disease 2019 (COVID-19) pandemic significantly impacted medical education. This study aimed to determine how COVID-19 affected students’ opportunity to practice core clinical skills across specialty rotations and their self-perceived proficiency at performing these.

**Methods:** Routinely administered surveys of fifth year medical student’ experiences and perceptions of medical training from 2016 to 2021 were analysed. Number of times core clinical skills were performed and self-perceived proficiency of each skill were compared pre- (years 2016-2019) and during-COVID (years 2020-2021).

**Results:** Data from 219 surveys showed a reduction in the opportunity to perform “a cervical screen test” (p<0.001), “a mental health assessment” (p=0.006), “assess the risk of suicide” (p=0.004) and “bladder catheterisation” (p=0.007) during-COVID. Self-reported skill proficiency was also less during-COVID for performance of: “a mental health assessment” (p=0.026) and “an ECG” (p=0.035).

**Conclusions: **The impact of COVID-19 on mental health skills was greatest, potentially due to a shift toward telehealth services and consequent reduced ability for students to engage in consultations. In a time of potential long-term change in the healthcare landscape, it is imperative to ensure adequate opportunity to practice all core clinical skills during medical training. Inclusion of telehealth earlier into the curriculum may benefit student confidence.

## Introduction

After its emergence in China, the novel coronavirus disease 2019 (COVID-19) was soon declared a global pandemic by the World Health Organisation in March 2020 ( World Health, 2020). Many countries worldwide quickly implemented public policies to reduce the spread of COVID-19 including: international/national travel restrictions; closures of workplaces, schools and public transport; public gathering cancellations/restrictions; stay-at-home requirements; contact tracing; mandatory vaccination and facial coverings ( Hale *et al.*, 2022).

COVID-19 has significantly impacted all aspects of society, including the education sector. Australian medical schools, like most schools across the globe, were required to rapidly transform and restructure medical education to comply with fast-changing government policies whilst maintaining a quality, valid, reliable curriculum. Online learning, digital teaching and modified assessments ( Heal *et al.*, 2022) were fast-tracked to ensure continuity of student education and clinical training.

The full impact of COVID-induced changes to medical education is yet to be quantified. International studies have shown that while some students have welcomed the flexibility of online learning, many felt the disruptions to the curriculum, particularly clinical placements, would negatively affect clinical competencies and learning quality ( Dost *et al.*, 2020; Huddart *et al.*, 2020; Martin *et al.*, 2022; Olmes *et al.*, 2021). In Australia, COVID-imposed restrictions resulted in reduced elective surgeries and emergency department presentations, and a move towards telehealth consultations for general practitioners and medical specialists in 2020 ( Australian Institute of Health & Welfare, 2021). These changes to health services are likely to have a flow-on effect on medical training programs and education, particularly clinical rotations which cannot be easily replaced by online or virtual environments.

The aim of the current study was to assess the effect of COVID-induced changes to healthcare services and medical education on students’ exposure to and ability to practice fundamental clinical skills and their self-perceived proficiency of each skill at the end of the fifth academic year in a rural setting.

## Methods

Fifth year medical students at James Cook University (JCU) undertake medical rotations across six core clinical disciplines as a part of standard medical training. Exit examinations are undertaken at the end of 5 ^th^ year, and the 6 ^th^ year of the course is considered to be a student internship. Surveys of student experiences and perceptions of medical training have been routinely administered to fifth year medical students since 2005. These are completed at the end of each academic year to allow students to highlight strengths and weaknesses in the program. Senior clinicians from each rotation nominated three clinical skills believed to be fundamental to the curriculum ( Table 1) and these were integrated into the survey. It should be noted that students were asked to rate the nominated clinical skill based on performance during the relevant rotation, rather than across the entire year e.g., performance of a mental health assessment only during the mental health rotation. Students indicated, using 5-point Likert scales, the number of times the identified fundamental clinical skills were performed in each rotation (1 = never, 2 = Once, 3 = Twice, 4 = three times, 5 = more than three times) and rated their self-perceived proficiency for each skill (1 = very poor, 2 = poor, 3 = indifferent, 4 = good, 5 = very good). This study analyses data from all surveys conducted from 2016 to 2021 (to allow comparison of pre-COVID to COVID data). No identifiable data information was collected to maintain student anonymity.

**Table 1.** Comparison of number of times clinical skills were performed during pre-COVID-19 and COVID-19 years.

		Number of times skill performed during core rotation				
Core discipline rotations	Clinical skill	Pre-COVID (2016-2019)		COVID (2020-2021)		p-value
		< 3	≥3	< 3	≥3	
Child and Adolescent Health	Examine a newborn	20	120	11	40	0.227
	Perform a developmental assessment	74	60	29	21	0.736
	Calculate medication dose for a child	100	35	37	14	0.833
Reproductive and Neonatal Health	Perform a cervical screening test	29	105	25	27	<0.001
	Examine a pregnant abdomen >32 weeks	3	130	3	49	0.225
	Identify foetal heart with Doppler	2	132	2	50	0.321
Mental Health	Perform a mental health assessment	15	120	14	37	0.006
	Assess the risk of suicide	34	101	24	27	0.004
	Establish rapport with a difficult patient	43	92	21	30	0.232
Adult Health - Medicine	Perform an ECG	86	49	25	27	0.051
	Take blood glucose using glucometer	60	75	20	30	0.588
	Perform venepuncture	45	90	14	37	0.442
Adult Health - Surgery	Take sample for blood gases	123	15	43	8	0.369
	Suture skin	60	71	22	29	0.746
	Perform bladder catheterisation	95	38	25	25	0.007
General Practice	Diagnose otitis externa on otoscope	30	48	18	20	0.361
	Perform skin excision	60	34	18	16	0.265
	Perform urinalysis	22	111	9	42	0.858

### Data analysis

Data were collated and analysed using Excel (Microsoft Office 365, v2202, 2022). Data on the number of times skills were performed in the associated rotation was not considered linear and therefore were condensed into two categories, less than three times, and three times or more. The decision for the cut-off point for this dichotomy was based on the pragmatic “See One, Do One, Teach One” teaching method traditionally used in medical training in which the three most optimal strategies for retaining information are employed i.e. discussion, practice and teaching ( Lalley & Miller, 2007). Hence, the minimum number of times a skill should be practiced with adequate supervision, guidance and critical feedback was considered three. Similarly, self-rated proficiency scores were categorised into “lacks proficiency” (inclusive of scores of very poor, poor, and indifferent) or “proficient” (scores of good and very good). Years 2016 to 2019 inclusive were considered “pre-COVID” and 2020 to 2021 as “COVID”.

The chi-square test was used to investigate the association between year of rotation (pre-COVID and COVID) and the categorical variables of number of times a skill was performed (less than three; three or more), and self-proficiency rating (lacks proficiency; proficient). Statistical significance was considered as *P* < 0.05.

***Ethical considerations.*** This study was approved by the James Cook University Human Research Ethics Committee (H5595 - approved 6 ^th^ May 2014, and H6921 – approved 8 ^th^ May 2017). Participants were informed that completion of the survey implied consent for the study, as per the ethical clearance stated above.

## Results

A total of 219 surveys were sent out to 5 ^th^ year medical students across the 6-year data collection period (pre-COVID n=150) of which 188 were returned (pre-COVID n=134) and analysed. The overall response rates pre- and during COVID were 89.3% and 78.3%, respectively.

The number of times students were able to perform four of the 18 identified fundamental clinical skills reduced during COVID. These skills were: “perform a cervical screen test” (p<0.001), “perform a mental health assessment” (p=0.006), “assess the risk of suicide” (p=0.004) and perform bladder catheterisation” (p=0.007) ( Table 1).

Despite a reduction in the four skills listed above, self-reported skill proficiency was less during COVID than the four years pre-COVID for two skills only: “Perform a mental health assessment” (p=0.026) and “Perform an ECG” (p=0.035) ( Table 2).

**Table 2.** Comparison of self-reported clinical skill proficiency during pre-COVID-19 and COVID19 years.

		Self-rated skill proficiency				
Core discipline rotations	Clinical skill	Pre-COVID (2016-2019)		COVID (2020-2021)		p-value)
		Not proficient	Proficient	Not proficient	Proficient	
Child and Adolescent Health	Examine a newborn	49	88	18	31	0.904
	Perform a developmental assessment	104	32	32	16	0.184
	Calculate medication dose for a child	104	32	30	18	0.061
Reproductive and Neonatal Health	Perform a cervical screening test	50	87	18	32	0.95
	Examine a pregnant abdomen >32 weeks	20	116	10	40	0.384
	Identify foetal heart with Doppler	17	120	6	44	0.94
Mental Health	Perform a mental health assessment	39	97	23	27	0.026
	Assess the risk of suicide	40	96	20	30	0.171
	Establish rapport with a difficult patient	60	76	26	24	0.339
Adult Health - Medicine	Perform an ECG	54	84	11	38	0.035
	Take blood glucose using glucometer	28	110	5	45	0.101
	Perform venepuncture	64	74	21	27	0.753
Adult Health - Surgery	Take sample for blood gases	116	17	38	11	0.109
	Suture skin	77	60	22	27	0.173
	Perform bladder catheterisation	88	46	30	19	0.578
General Practice	Diagnose otitis externa on otoscope	77	90	24	28	0.995
	Perform skin excision	81	83	31	29	0.763
	Perform urinalysis	16	119	6	44	0.978

## Discussion

This study showed that changes due to COVID-19 were correlated with a reduction in the number of times students were able to perform four of their 18 (22%) fundamental clinical competencies. There was a reduction in self-perceived skill proficiency in two of the 18 skills, and in only one clinical skill, completion of a mental health assessment, was there an alignment, with a reduction in both measures.

Self-perceived proficiency is subjective, and prone to many sources of variation, which may explain the lack of alignment between measures. Performance of all the clinical skills are impacted by variables causing potential confounding, such as temporal changes in the use and appropriateness of the skill, changes in co-ordinating staff, and changes in student cohorts. A reduction in the frequency of bladder catheterisation may have been a reflection of reduced elective surgery due to COVID-19. A reduction in the number of times performing electrocardiograms (ECGs) may have been a reflection in the reduction in normal inpatient care, although this skill could be easily simulated. 

Of the four clinical skills performed with reduced frequency in the covid period, cervical screening is the easiest to explain. The data collection period encompassed a period of transition in the National Cervical Cancer Screening Program. From December 2017, the 2-yearly Pap test was phased out and replaced by the 5-yearly cervical screening program ( Australian Government Department of Health, 2022), with 2020 the first year impacted by this change. The number of women presenting for cervical screening was further reduced from April when COVID-19 restrictions were implemented ( Australian Government Department of Health, 2022).

It is estimated the prevalence of anxiety and depression worldwide has increased 25% in the first 12 months of the COVID-19 pandemic ( World Health Organization, 2022). This is reflected in Australian data that compared use of mental health services in September 2020 to the same period in 2019. This showed a 14.5% increase in the number of Medicare Benefits Schedule (MBS) subsidised mental health services delivered, a 6.0% increase in mental health-related prescriptions, and greater than a 14% increase in calls to support organisations (Lifeline: 15.6%; Kids Helpline: 14.3%; Beyond Blue: 21.3%) ( Australian Institute of Health & Welfare, 2021). However, although there was an increased burden of mental health, and an increased delivery of mental health care in general practice, it is likely that the exposure to mental health for students during their hospital based mental health services rotations were reduced.

The decrease in students completing mental health assessments and suicide risk assessments may have been due to a number of factors. First, nationwide restrictions were introduced including social distancing measures and limits on the number of people permitted in hospitals and medical clinics. These are known to impact clinical session time, patient consultation access and patient interaction in rural clinical settings ( Hoang *et al.*, 2022). Fewer inpatients in the mental health rotation will have impacted student experience.

Second, it is likely that the proportion of hospital outpatient and community health mental health consultations delivered with telehealth increased. Australian studies have shown that prior to COVID-19, students had limited exposure to telehealth during clinical medical training as it was not a standard inclusion in the medical curriculum ( Edirippulige *et al.*, 2022; Pit *et al.*, 2021). Students may have found it more difficult to engage with telehealth consultations due to the somewhat detached nature of delivery and the inability to read non-verbal communication present in face-to-face services, especially in consultations conducted via telephone. Pit *et al.* identified telephone consultations could also be problematic if not conducted via speakerphone as students would only hear the doctor’s side of the conversation, and that telehealth was not always appropriate for discussing sensitive issues such as those associated with mental health ( Pit *et al.*, 2021).

Third, students’ mental health and well-being may have impacted their self-reported proficiency and participation in performing mental health clinical skills, and lack of this data may be considered a limitation. Many causes of COVID-related stressors have been identified including transition to online learning and assessment, relocation and personal and family health and finances ( O’Byrne *et al.*, 2021). Psychological distress (stress, anxiety or depression) is associated with poor self-perception of performance as well as impaired concentration levels ( Chandavarkar *et al.*, 2007; Yamada *et al.*, 2014). It is also reasonable to infer that students who were vulnerable and experiencing symptoms of mental health disorders may have avoided practising and studying this aspect of their syllabus, as part of a maladaptive avoidance coping mechanism ( Ball & Gunaydin, 2022). A previous study reported increased stress and anxiety in a large percentage (84.1%) of medical students surveyed due to disruptions in their medical education from the COVID-19 pandemic ( Harries *et al.*, 2021). This was supported by another survey that showed mental health was negatively impacted by pandemic-induced curriculum changes in more than half of medical students (68%) at an Australian university ( Lyons *et al.*, 2020). Thus, students’ mental health and wellbeing is a key element that may have contributed to the reduction in performance of mental health clinical skills combined with a drop in self-reported proficiency. The setting of health care in general has shifted somewhat towards telehealth, a proportion of which will undoubtedly remain after the pandemic. It is important to emphasise that students must have the ability to participate and practice their skills when consultations are shifted to telehealth and that their skills in this advancing field be built upon early in their medical training. Whilst this is important across all clinical rotations, particular attention should be given to mental health as this has been identified as one specialty in which students perceive to have deficits in opportunity and proficiency. It is also imperative that health professionals and medical educators are aware of the increase in mental health concerns amongst medical students, particularly during the pandemic, as this may to contribute to poor clinical learning experiences and outcomes.

Ironically, at a time in which mental health is a major global concern, medical students were least able to practice the skill of performing a mental health assessment within their mental health rotation and also felt their proficiency was lower in this area than students in the preceding four years before COVID-19 began. The number of times they were able to assess the risk of suicide was also reduced in the two COVID-affected academic years. Thankfully, despite dire warnings of a COVID-19 suicide tsunami, with the exception of Japan ( Okada *et al.*, 2022), suicide rates across the world have remained stable during the pandemic ( Tandon, 2021). While this is encouraging, suicide rates in general remain high and it is the fourth leading cause of death in people aged 15–29 years ( World Health Organization, 2021).

A limitation of this study is its lack of generalisability to other Australian regions as it was conducted in a rural North Queensland setting. COVID-19 restrictions in Queensland were less severe and shorter-lived than those experienced in southern states such as New South Wales and Victoria. With fewer and shorter lockdowns, especially in North Queensland, it is possible the impact of COVID-19 on medical clinical training was less than southern states.

## Conclusion

Medical student clinical training was negatively impacted by restrictions imposed during the COVID-19 pandemic. The number of times students were able to perform or practice four of the 18 fundamental clinical skills was reduced compared to students on clinical rotations in the 4 years prior to COVID-19. Their self-perceived proficiency was also reduced for two skills (“Perform a mental health assessment” and “Perform an ECG”). Skills related to mental health assessment were most affected. With the increased focus on mental health since the beginning of the pandemic, it is imperative that students are provided sufficient opportunity to practice and build confidence in core skills in this specialty.

## Data Availability

Data can be requested from James Cook University and is held in their repository. All reasonable requests for collated data will be considered on a case by case basis. The data forms part of the Universities formal course evaluation and contains the names of individual academic staff members and cannot be shared for privacy reasons. A redacted version may be made available upon reasonable request to the corresponding author ( clare.heal@jcu.edu.au).
