# Supplementary material for: The effect of COVID-19 on medical student clinical skill practice and self-perceived proficiency
Source: MedEdPublish (2016). 2023 Apr 26;13:10. Originally published 2023 Feb 8. [Version 2] doi: 10.12688/mep.19478.2 (PMC10331848; doi:10.12688/mep.19478.2)
Supplement: Peer review history

# Peer review: Reviewer response for version 2

**DOI:** 10.21956/mep.21057.r33180

**Published:** 2023-05-18

**Authors:** Anita Samuel

**Affiliations:** 1 Department of Medicine, Center for Health Professions Education, Uniformed Services University, Bethesda, MD, USA

Thank you for addressing the concerns raised.

Have any limitations of the research been acknowledged?

Partly

Is the work clearly and accurately presented and does it cite the current literature?

Yes

If applicable, is the statistical analysis and its interpretation appropriate?

Yes

Are all the source data underlying the results available to ensure full reproducibility?

Partly

Is the study design appropriate and is the work technically sound?

Yes

Are the conclusions drawn adequately supported by the results?

Yes

Are sufficient details of methods and analysis provided to allow replication by others?

Yes

Reviewer Expertise:

Areas of research include curriculum development, online education, distance education, educational technology

I confirm that I have read this submission and believe that I have an appropriate level of expertise to confirm that it is of an acceptable scientific standard.

# Peer review: Reviewer response for version 2

**DOI:** 10.21956/mep.21057.r33181

**Published:** 2023-04-27

**Authors:** Jane Smith

**Affiliations:** 1 Faculty of Health Sciences and Medicine, Bond University, Robina, QLD, Australia

Have any limitations of the research been acknowledged?

Yes

Is the work clearly and accurately presented and does it cite the current literature?

Yes

If applicable, is the statistical analysis and its interpretation appropriate?

I cannot comment. A qualified statistician is required.

Are all the source data underlying the results available to ensure full reproducibility?

Yes

Is the study design appropriate and is the work technically sound?

Yes

Are the conclusions drawn adequately supported by the results?

Yes

Are sufficient details of methods and analysis provided to allow replication by others?

Yes

Reviewer Expertise:

clinical medical education and assessment plus general practice

I confirm that I have read this submission and believe that I have an appropriate level of expertise to confirm that it is of an acceptable scientific standard.

# Peer review: Reviewer response for version 1

**DOI:** 10.21956/mep.20871.r33000

**Published:** 2023-04-03

**Authors:** Jane Smith

**Affiliations:** 1 Faculty of Health Sciences and Medicine, Bond University, Robina, QLD, Australia

This research demonstrates and quantifies a decline in clinical skills training,and self perceived competencies in those clinical skills, experienced by medical students in North Queensland during the pandemic. There are many anecdotal reports from medical schools of students and graduates nor achieving the same levels of clinical training nor achieving the level of competencies as pre-pandemic. This study shows a difference in some of these skills in a part of the world less affected than most.

 It would have been good to see this point emphasised more but there is not much literature about this out there.

 I believe the statistical analysis appropriate but I am not a statistician.

 The data summary is provided with number of surveys and return rate, also available on request, which would enable a repeat of the analysis

Have any limitations of the research been acknowledged?

Yes

Is the work clearly and accurately presented and does it cite the current literature?

Yes

If applicable, is the statistical analysis and its interpretation appropriate?

I cannot comment. A qualified statistician is required.

Are all the source data underlying the results available to ensure full reproducibility?

Yes

Is the study design appropriate and is the work technically sound?

Yes

Are the conclusions drawn adequately supported by the results?

Yes

Are sufficient details of methods and analysis provided to allow replication by others?

Yes

Reviewer Expertise:

clinical medical education and assessment plus general practice

I confirm that I have read this submission and believe that I have an appropriate level of expertise to confirm that it is of an acceptable scientific standard.

# Response

**Published:** 2023-04-18

**Authors:** Leanne Hall

**Affiliations:** James Cook University, Australia

We thank the reviewer for her time in reviewing our paper and the positive comments.

# Peer review: Reviewer response for version 1

**DOI:** 10.21956/mep.20871.r33020

**Published:** 2023-04-03

**Authors:** Anita Samuel

**Affiliations:** 1 Department of Medicine, Center for Health Professions Education, Uniformed Services University, Bethesda, MD, USA

This article reports the findings of a study conducted at a rural university in Australia. This study explores how social distancing regulations during the COVID-19 pandemic affected medical students during their clinical rotations. The authors look at 22 clinical skills and present changes to how many times these skills were performed and students' self-reported skill proficiency. The study findings identify only 4 statistically significant clinical skills that were performed fewer times.

 Furthermore, students' self-reported proficiency was reduced in only one clinical skill. 

 There are a few points that need clarification. 

- While students showed a decrease in the number of times they performed 4 clinical skills, they only showed a decrease in self-reported proficiency of one of the skills. It would be helpful to explore this difference. What implications does this difference have for teaching clinical skills? 
- This study provides a snapshot of what happened during a specific point in time - COVID-19. The interest and implications of this study are very bound by this context. The authors need to move this study beyond the COVID context and consider larger implications for practice. Without this, the study leaves the reader questioning the significance of this study. 
- The only limitation identified by the authors is that the study was conducted in a rural Australian setting and might not be generalizable. However, there are other limitations as well. For example, the reliance on self-reported proficiencies and the COVID-19 context. 

Have any limitations of the research been acknowledged?

Partly

Is the work clearly and accurately presented and does it cite the current literature?

Yes

If applicable, is the statistical analysis and its interpretation appropriate?

Yes

Are all the source data underlying the results available to ensure full reproducibility?

Partly

Is the study design appropriate and is the work technically sound?

Yes

Are the conclusions drawn adequately supported by the results?

Yes

Are sufficient details of methods and analysis provided to allow replication by others?

Yes

Reviewer Expertise:

Areas of research include curriculum development, online education, distance education, educational technology

I confirm that I have read this submission and believe that I have an appropriate level of expertise to confirm that it is of an acceptable scientific standard, however I have significant reservations, as outlined above.

# Response

**Published:** 2023-04-18

**Authors:** Leanne Hall

**Affiliations:** James Cook University, Australia

This article reports the findings of a study conducted at a rural university in Australia. This study explores how social distancing regulations during the COVID-19 pandemic affected medical students during their clinical rotations. The authors look at 22 clinical skills and present changes to how many times these skills were performed and students' self-reported skill proficiency. The study findings identify only 4 statistically significant clinical skills that were performed fewer times. Furthermore, students' self-reported proficiency was reduced in only one clinical skill. There are a few points that need clarification. While students showed a decrease in the number of times they performed 4 clinical skills, they only showed a decrease in self-reported proficiency of one of the skills. It would be helpful to explore this difference. What implications does this difference have for teaching clinical skills?

 Response: Thank you for your time in reviewing our paper and feedback which allows us to improve the article content. There was a reduction in 2 of the proficiencies, but only one of these was accompanied by a reduction in frequency of skill performance. It is difficult to postulate the implications for teaching as we move back into an environment that more resembles pre-COVID medical training. We have attempted to address this comment by adding the following text to the discussion. “The teaching of mental health assessment skills may need to be reassessed in a post-COVID landscape where non-face-to-face methods of clinical delivery of mental health are likely to be retained, at least in part. Simulation may have a role in teaching of mental health skills, as well as reassessing how to engage medical students in telehealth consultation”.

 This study provides a snapshot of what happened during a specific point in time - COVID-19. The interest and implications of this study are very bound by this context. The authors need to move this study beyond the COVID context and consider larger implications for practice. Without this, the study leaves the reader questioning the significance of this study.

 Response: The idea of this paper was to identify the impact of changes at a specific timepoint, i.e. during COVID-19 We have attempted to address this in part with the comment above on using the experience of COVID-19 in which face-to-face training was not always possible to help direct changes in teaching where applicable. It is difficult to simulate many clinical skills despite advances in simulation techniques therefore current methods of medical training are most likely required to be retained into the future both to enhance clinical skill ability and to allow students to feel more confident in their abilities to perform them in clinical practice.

 The only limitation identified by the authors is that the study was conducted in a rural Australian setting and might not be generalizable. However, there are other limitations as well. For example, the reliance on self-reported proficiencies and the COVID-19 context.

 Response: We have included other limitations throughout the discussion. E.g. “Third, students’ mental health and well-being may have impacted their self-reported proficiency and participation in performing mental health clinical skills, and lack of this data may be considered a limitation” and “Self-perceived proficiency is subjective, and prone to many sources of variation, which may explain the lack of alignment between measures.”. We have amended the text to add other limitations as follows. “First, as mentioned above, self-perceived proficiency is a subjective measure of skill performance that can be affected by a myriad of factors pertaining both to the student personally and their learning environment. An objective measure of skill performance (e.g. clinical grading for each of the identified skills) would have provided additional information and served as a good comparator to self-proficiency. This was not possible as the surveys were made anonymous to increase student response rates and gain more honest feedback about performance, therefore negating our ability to cross-reference subjective and objective measures of performance”.
